# A resilient workforce: patient safety and the workforce response to a cyber-attack on the ICT systems of the national health service in Ireland

**DOI:** 10.1186/s12913-023-10076-8

**Published:** 2023-10-17

**Authors:** Gemma Moore, Zuneera Khurshid, Thérèse McDonnell, Lisa Rogers, Orla Healy

**Affiliations:** 1 Health Service Executive, National Quality and Patient Safety Directorate, Dublin, Ireland; 2 UCD IRIS Centre, School of Nursing, Midwifery and Health Systems, University College Dublin, Dublin, Ireland; 3 Improvement Academy, Bradford Institute for Health Research, National Health Service, Bradford, England

**Keywords:** Resilience, Health systems, Health service staff, Cyber-attack, Patient safety

## Abstract

**Background:**

In May 2021, the Irish public health service was the target of a cyber-attack. The response by the health service resulted in the widespread removal of access to ICT systems. While services including radiology, diagnostics, maternity, and oncology were prioritised for reinstatement, recovery efforts continued for over four months. This study describes the response of health service staff to the loss of ICT systems, and the risk mitigation measures introduced to safely continue health services. The resilience displayed by frontline staff whose rapid and innovative response ensured continuity of safe patient care is explored.

**Methods:**

To gain an in-depth understanding of staff experiences of the cyber-attack, eight focus groups (n = 36) were conducted. Participants from a diverse range of health services were recruited, including staff from radiology, pathology/laboratories, radiotherapy, maternity, primary care dental services, health and wellbeing, COVID testing, older person’s care, and disability services. Thematic Analysis was applied to the data to identify key themes.

**Results:**

The impact of the cyber-attack varied across services depending on the type of care being offered, the reliance on IT systems, and the extent of local IT support. Staff stepped-up to the challenges and quickly developed and implemented innovative solutions, exhibiting great resilience, teamwork and adaptability, with a sharp focus on ensuring patient safety. The cyber-attack resulted in a flattening of the healthcare hierarchy, with shared decision-making at local levels leading to an empowered frontline workforce. However, participants in this study felt the stress placed on staff by the attack was more severe than the cumulative effect of the COVID-19 pandemic.

**Conclusions:**

Limited contingencies within the health system IT infrastructure - what we call a lack of system resilience - was compensated for by a resilient workforce. Within the context of the prevailing COVID-19 pandemic, this was an enormous burden on a dedicated workforce. The adverse impact of this attack may have long-term and far-reaching consequences for staff wellbeing. Design and investment in a resilient health system must be prioritised.

**Supplementary Information:**

The online version contains supplementary material available at 10.1186/s12913-023-10076-8.

## Background

Health care professionals are the resource that underpin the delivery of safe and high-quality care in a resilient health system. However, resilient healthcare does not focus on the individual’s capacity to cope but rather on what enables the workers, team, and unit or organisation to adapt and cope effectively in different situations [1]. While definitions and concepts of health system resilience differ substantially throughout the literature, all have a common foundation: they regard resilience as the degree of change a system can undergo while maintaining its functionality [2, 3]. Health system resilience is not a single dimension but rather an emergent property of the health system as a whole [4], and requires a strong and committed health workforce, characterised by healthcare personnel who show up for work that might be difficult and, in certain contexts, dangerous [4].

Health system resilience has also been defined as the capacity to absorb, adapt, and transform when exposed to a shock, such as a pandemic, natural disaster or armed conflict and still retain the same control over its structure and functions [5]. While continuing to respond to the challenges presented by the COVID-19 pandemic, the Irish public health system experienced a significant further shock to the delivery of healthcare when access to ICT systems across most services nationally ceased. On Friday, 14th May 2021, the Health Service Executive (HSE) of Ireland was the target of a cyber-attack, a criminal infiltration of the HSE’s IT systems using Conti ransomware. In response, the HSE invoked its Critical Incident Process, leading to the decision to switch off all HSE ICT systems and disconnect the National Healthcare Network (“NHN”) from the internet [6]. The HSE is responsible for the national public health service in Ireland, serving a population of 5.1 million people. As the largest employer in the country, the HSE employs over 130,000 staff directly and indirectly, with over 70,000 devices such as laptops and PCs in use [6]. The public health service is provided at approximately 4,000 locations, including 54 acute hospitals. Invoking the Critical Incident Process immediately resulted in healthcare professionals losing access to all HSE provided IT systems - including patient information, clinical care and laboratory systems. Non-clinical systems such as financial systems, payroll and procurement systems were also lost. Normal communication channels such as email and networked phone lines were no longer available [6]. Therefore, this attack and the sudden removal of ICT system access presented a monumental challenge to staff tasked with continuing to deliver safe healthcare.

The impact of this attack on the Irish health system is illustrated by a framework proposed by Thomas et al. (2020) which sets resilience within the context of a 4-stage shock: preparedness; shock onset and acting rapidly; managing impact to preserve health system access and quality; and recovery and learning [7]. While the HSE was not prepared for this cyber-attack [6], it did respond rapidly to limit the impact of the attack on IT systems nationally. Integrated governance structures were quickly established to oversee and expedite the clinical and operational response. Health services including patient administration systems, radiology, diagnostics, maternity, and oncology were prioritised to advance the resumption of systems [8]. However, recovery efforts continued for over four months, a timescale far greater that initially expected [6], leaving many staff to manage the impact and preserve health system access and quality over a sustained period. The risk of cyber-attacks on health systems amplified during the COVID-19 pandemic due to increased remote work and reliance on virtual methods of care delivery [9, 10]. In 2020 alone, a number of healthcare organisations across a range of countries experienced a cyber-attack, including Brno University Hospital in the Czech Republic, Hammersmith Medicines Research Group and Babylon Health in the UK, and Paris Hospital Authority (APHP) in France [10]. Such attacks caused disruption to appointment systems, patient records, imaging and surgical services, and medical devices. Organisations need to adopt a “security culture” to reduce the risk of these attacks [10] and recent research has focused on the technical reasons that enabled these attacks and mitigation strategies that may reduce the likelihood of future attacks [11, 12]. However, there has been little focus on the implications of such attacks on those tasked with continuing service delivery in the absence of core IT and communication systems. This study contributes to a greater understanding of the impact on staff and service delivery by describing the response of staff within the Irish health service to the loss of ICT systems due to the cyber-attack in May 2021, and the risk mitigation measures and contingencies introduced to safely continue health and social care services. The resilience of healthcare staff who quickly adapted and innovated to ensure continuity of service and patient safety is explored.

## Methods

The HSE National Quality & Patient Safety Directorate commissioned a mixed-methods research study to understand the clinical impact of the Conti cyber-attack on patient safety [8]. This paper presents the findings of one aspect of this overall study, a qualitative analysis of focus groups exploring the experiences of staff working in acute, maternity, and community settings.

### Recruitment

Different settings included in the study were a large university hospital, a maternity teaching hospital, and various sites from one Community Healthcare Organisation (CHO). In consultation with the relevant Heads of Service for Quality Safety and Service Improvement, representatives from services in these sites most affected by the cyber-attack were invited to participate in focus groups to share their experiences and learning. Purposeful sampling identified clinicians, executive managers, scientists and other administrative and support staff who were invited to participate voluntarily. This included radiology, pathology/labs, radiotherapy, maternity, primary care dental services, health and wellbeing, COVID testing, older person’s care, and disability services. A detailed study information sheet was provided (Appendix File 1) to potential participants and staff self-selected to participate. Sample size was determined in consultation with Quality and Safety, hospital and community service managers. Data saturation was identified early during analysis which confirmed the adequacy of the sample size.

### Data collection & analysis

Using an in-depth semi-structured topic guide (Appendix File 2), eight focus groups with 36 participants were conducted over a four-week period in September and October 2021, with each focus group lasting approximately 60 min. Focus groups were conducted by two experienced qualitative researchers virtually using online platforms (MS Teams and Cisco WebEx), depending on the preference of the participants due to Covid restrictions. Informed consent was sought, and participants were given the opportunity to ask questions and voice any concerns and asked to sign a consent form prior to the start of the focus groups (Appendix Files 3). The eight focus groups were conducted with staff working in clinical, business management, scientists, administrators, and information systems roles (Table 1).

**Table 1:** Focus group details

Care Area	Focus group	Service type	Number of participants
Community	Focus group 1	Dental Services	2
	Focus group 2	Health and Wellbeing and COVID testing	4
	Focus group 3	Disability	3
	Focus group 4	Social Care – Older Persons	2
Acute	Focus group 5	Radiotherapy	8
	Focus group 6	Radiology	3
	Focus group 7	Laboratory Services	4
Maternity	Focus group 8	Maternity services	10
**Total participants**			**36**

Thematic Analysis, as outlined by Braun and Clarke [13], guided the analysis structure. This process involved repeatedly reading the data, generating initial codes, and developing, refining, and naming broader themes. Rather than applying a prescriptive list of codes, an inductive approach to coding was chosen to ensure the themes generated strongly reflected the data collected. One researcher (TMD) analysed the complete dataset, while a second experienced qualitative researcher (LR) double coded a random subset of the transcripts (n = 3) to ensure consistency. Themes were further reviewed and grouped through discussions with the research team. NVivo 12 software supported the analysis process.

The inductive analysis of the transcripts from the eight focus groups (Table 1) generated a number of initial codes representing patterns in the data. These patterns were grouped under headings or potential themes. For example, an initial theme of *Mitigations* included codes for challenges, facilitators and new approaches. Codes were further reviewed to ensure similar data were grouped together while each theme remained distinct. Finally, a title that most precisely reflected the underlying data was collaboratively agreed for each theme. To illustrate, the final theme *Staff Commitment Facilitating Service Continuity* (Table 1) was initially labelled *Mitigations* by *TMD* and *Response Adaptions* by *LR*. The refinement of this theme was conducted collaboratively by both *TMD* and *LR* in conjunction with two further members of the research theme, *GM* and *ZK*.

## Results

**Fig. 1:**
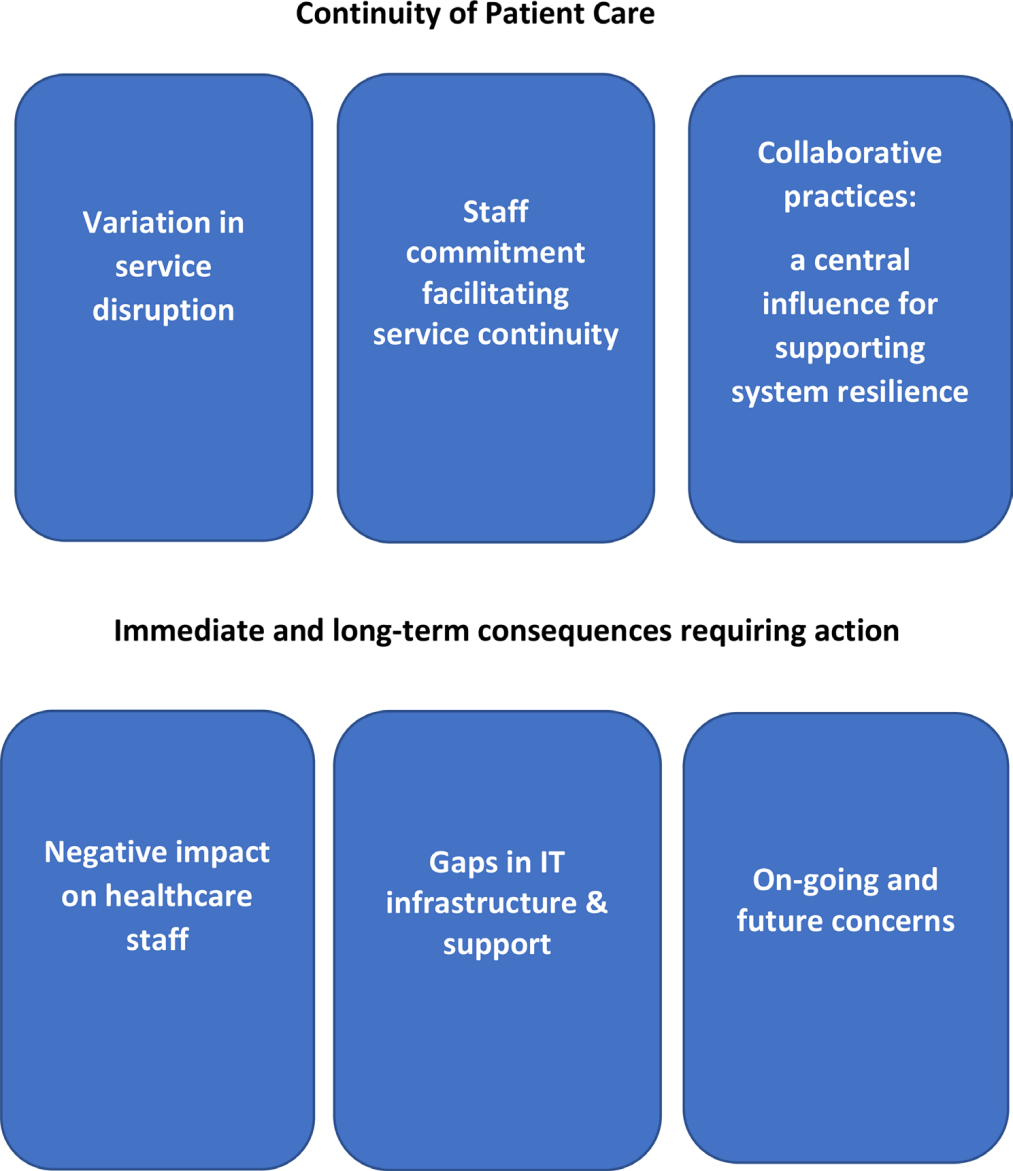
Themes

The inductive analysis generated six key themes within two broader categories: (1) continuity of patient care and (2) immediate and long-term consequences requiring action (Fig. 1).

### Continuity of patient care

Healthcare staff worked diligently to ensure the continuation of services, at least at a basic level, while ICT systems remained inaccessible. Three themes were identified that explain the extent of the disruption to services and how the nature of the response by staff supported the resilience of the health system.

### Variation in service disruption

The impact of the cyber-attack varied across services depending on the reliance on software, the type of care being offered, and local IT support. The cyber-attack’s duration was unprecedented and backup systems contained basic patient data for the last 7–15 days, which quickly became irrelevant. Participants in acute and maternity services were completely reliant on electronic methods and were therefore more severely impacted:

“*our emails and other files and everything was gone. But they were the least of our worries now to be honest. So our lab information system was gone.”* (Participant A12).

Patient information systems were not accessible, leaving clinical staff to treat patients without knowing their full history. Clinical care and laboratory systems could not be accessed, with clinicians *“in the flick of a switch”* (Participant A12) unable to carry out routine diagnostic procedures. There was a significant impact on radiotherapy services, with cessation of radiation treatment:

*“Most critically the patients who were on treatment having radiotherapy there was absolutely no way of continuing their treatment .before we even got to that point. there was no way of even identifying who they were to contact them not to come to their treatment.”* (Participant A3).

However, the effect of the cyber-attack was less severe for those working outside the acute services. In particular, the impact was *“negligible”* (Participant C7) for services still reliant on paper-based records due to low levels of computerisation. One unexpected outcome from the community care focus groups was that in the absence of ICT systems, staff found more time to spend with service users in residential homes: *“you actually had people who could sit and talk to residents”* (Participant C9).

### Staff commitment facilitating service continuity

As the cyber-attack impacted their normal systems and practices, staff had to adapt to the challenge, implement workarounds and refine mitigations rapidly to ensure continuity of services:

*“So I suppose we mitigated as best we could with the resources we had to try and keep the patients as safe as possible, and that involved really lateral thinking, like thinking outside the box.”* (Participant A7).

Staff held regular, often daily, in-person meetings, which previously had been limited due to the prevailing pandemic. Staff took the initiative “*to react and respond”* (Participant C10) and made decisions locally. Solutions involved developing manual paper-based systems and forms, which required additional training by more experienced healthcare professionals as many staff members had no experience of recording on paper, having always worked within a computerised environment. Non-digital solutions included returning to outdated methods, with radiologists using “*printing film*” (Participant A11) and “*carbon copy”* (Participant A10). Common mitigations across all services included setting up Gmail accounts in the absence of HSE email, using personal phones and WhatsApp, conference calls, and using the postal service.

The lack of patient information systems meant staff were not aware of who was due into the service and patients could not be individually contacted before attending the hospital. Therefore, notices were placed on external communication/websites requesting patients to phone in advance of scheduled appointments. Staff also reviewed the urgency of appointments and non-urgent appointments were postponed:

*“So what we did was we tried to stratify patients in terms of risk and anyone who was deemed low risk and an urgent appointment wasn’t required, their outpatient appointments were then postponed until such time as we had all the relevant details.”* (Participant M3).

Clinicians were forced to reconstruct very high dose radiotherapy relying on their recall, *“we literally had only our memories to rely on”* (Participant A6), to ensure the continuation of these critical treatments. Private hospitals were engaged to provide treatments, in particular to allow oncology patients to continue time-critical treatment. As priority systems began to return and many of these workarounds were no longer needed, staff worked tirelessly to ensure patients received much needed treatments:

*“When we did get the service up and running…we’re normally a Monday to Friday service, we treated every weekend for a number of weekends to try and compensate for the gaps…anyone who had a gap in their treatment, they got treated every weekend for their remaining treatment”.* (Participant A3)

Many of the workarounds introduced during this period were unwound once IT systems and communications were restored. However, many staff had a greater appreciation of the importance of maintaining accessible records, “*we always have a copy of {the} chart here now available in case we ever needed anything*”. (Participant A5), and the need to ensure such records are secure:

*“We need to have the system in place that we can make sure that we have the details of people that are safely kept as well, not off the laptop or anything…they’re stored properly, they’re maintained properly”. (Participant C4)*

Participants also note that the need for more direct communication has led to improved relationships between teams:

“*we’ve built some relationships with people and other teams around the hospital that wouldn’t have been in existence before*”. (Participant A7)

### Collaborative practices: a central influence for supporting system resilience

The cyber-attack resulted in a flattening of the hierarchy, with shared decision-making evident at local levels. This heightened autonomy led to an empowered frontline workforce. One participant described an “*equal platform*”, “*hierarchy was gone*” (Participant C6), which facilitated staff to speak up:

*“Yeah, and people were in pushing ideas, you know? So that there was a good yeah kind of collaboration of people, saying can we try this? and you know, in many cases the people on the floor as always had the solution.”* (Participant A10).

Participants reflected positively on how staff came together as a team, with communication playing a critical role in the success of the cyber-attack response. When asked what the key learning had been from this experience, one participant responded: *“teamwork, teamwork, teamwork and communication.”* (Participant A10). While communication underpinned the success of mitigations introduced, staff understood the need for flexibility due to the evolving situation:

*“There would have been an update as to where we were that morning and OK that could have changed by evening and, if necessary, there was an evening meeting and then that was fed back to I suppose the staff on the ground.”* (Participant A14).

However, while effective collaboration at local level made staff feel empowered to respond, participants identified several gaps in top-down communication during the cyber-attack. Communication nationally to local services was described as “*far from superior”* (Participant M4). There was frustration that the protracted nature of the disruption to health services was not communicated to the public: *“whether it wasn’t communicated or they weren’t picking up on it in the media”* (Participant A12), leaving local management feeling unsupported. There was also significant concern that the clinical risk posed by the removal of systems was not understood at the national level:

*“I don’t think there was the understanding there that our clinical risk was so great because we had absolutely no histories. All of the other hospitals had paper charts, whereas in the acutes and everywhere, we had absolutely nothing”* (Participant M4).

However, participants working on teams with local staff members attending national meetings were more positive about top-down communication, describing these links as *“valuable”* (Participant A6).

### Immediate and long-term consequences requiring action

Across all focus groups, healthcare staff raised concerns about the short and long-term consequences of the cyber-attack. Three themes were identified which reflect the impact of the response to the cyber-attack on employee wellbeing, shortcomings in the IT infrastructure and support, staff concerns about regaining productivity and the longer-term consequences of decisions made and manual processes implemented in the aftermath of the attack.

### Negative impact on healthcare staff

The dedication of staff in response to the cyber-attack enabled the continuity of patient care in extremely difficult circumstances. Health service staff had just come through four waves of COVID-19 when the cyber-attack occurred, with participants reporting that staff continued to experience high levels of stress, anxiety, and uncertainty as they dealt with concurrent challenges of the COVID-19 pandemic and the cyber-attack. Clinical decision-making without the support of systems and data left many clinicians working very long hours and deeply concerned about the choices they we forced to make:

*“We were putting in 16 plus hour days, significantly more I think at the beginning, and then the uncertainties that impacted that. So, I think that is all I have to say about that. I will say I still wake up at 4 in the morning in a cold sweat about what we did, but we had to do it.”* (Participant A6).

Participants expressed concern that the health system may be on the brink of a staff mental health crisis and the risk of a staff “*exodus”* (Participant A6) from the healthcare service. One participant commented that the current level of sick leave by staff *“has gone through the roof”* (Participant A3), with an increase in staff reporting *“mental health issues”* (Participant A3).

Concern was expressed that senior staff within the health service did not appreciate the work done locally by management and staff to respond to the situation. Participants also noted a desire for further acknowledgement of the resilience, altruism, and adaptability displayed by healthcare staff.

### Gaps in IT infrastructure & support

The cyber-attack has drawn attention to the lack of investment in IT infrastructure across all services. Participants explained how they had expressed concern about poor ICT systems over long periods of time, with one senior staff member working within community services commenting that “*we need to do something serious nationally in our approach to IT*” (Participant C1), while another senior staff member, also working within community services, commented:

*“There is a big gap if you look at a comparable organisation in the private sector - like their IT systems or their access is light years ahead of ours.”* (Participant C7).

While ICT systems are more advanced in the acute and maternity services, participants across these services also expressed concern about lack of investment. Old ICT systems in use throughout the health system are more vulnerable to cyber-attack, with one participant working in a laboratory setting commenting that “*we are Windows 7 based and we’d be hoping to move to Windows 10*” and further commented that “*the speed at which the IT is replaced within the HSE is a big problem”* (Participant A13). There was also a general perception that there is a lack of planning to address this deficit nationally: “*planning for that nationally, it doesn’t seem to be there”* (Participant A13). Participants reported a lack of trust in the ability of IT systems to protect against future events. While some services had backup systems in place, these backups were designed for shorter ICT outages and, due to the protracted nature of the event, the backups quickly became ineffectual.

Staff expressed frustration with IT support, “*I don’t think we have enough IT support locally or nationally, it’s all extremely frustrating*” (Participant C1). This frustration particularly related to the support provided through national helpdesks, but participants also mentioned delays in equipment procurement and set-up of new starters which all adversely impacted care delivery in the aftermath of the cyber-attack: “*the service level is bad to start with, but it’s way worse since the cyber-attack*” (Participant C1). Participants felt local IT staff on-site were needed to support their systems. The teams that had close relationships with their local IT support found it beneficial in understanding the emerging situation throughout the cyber-attack:

*“there’s a ICT department in X hospital which would be involved in PC and hardware replacement and we would have close ties and liaise with them and discuss any national information that they might have fed through their chief and would have a close link in with their line manager as well, so we were all working very closely to hope to try and get the best possible outcomes as quickly as possible, but also as safely as possible each way along, each step along the road”.* Participant A13.

### On-going and future concerns

Focus groups were conducted 4 to 5 months after the cyber-attack, yet all participants noted that the effects of the cyber-attack were on-going. Performance issues with core systems and lack of remote access to computers for some staff has impacted productivity:

*“You know everybody thinks we’re back to normal and actually a week later it kind of dropped off the news and stuff, but we were really suffering, and we continue…our productivity is very poor still from a digital point of view […]…so yeah, that’s our estimate. Yeah, 30% productivity down on the digital side still”.* (Participant A9)

Participants commented that cyber-security measures employed in response to the cyber-attack also resulted in systems becoming slower, less accessible, with an increase in equipment downtime as external support providers, such as engineers, could no longer remotely access equipment in many instances.

Staff were also concerned that the cyber-attack impacted the trust of patients and service-users. The adverse impact of the cyber-attack on waiting lists, already problematic due to the impact of the pandemic, added a further challenge to staff striving to deliver a quality service for patients:

*“We have been trying in our hospital to work on waiting lists and try and improve the quality and the timeliness of the service that we provide to our patients. COVID impacted on this, but the cyber-attack impacted even more on it and there’s nothing out there for our patients.”* (Participant M3).

Clinical staff also suspected that they will not be fully aware of the impact of mitigations on patients treated during this period for a long time to come. Manual workarounds developed were prone to risks such as redundancy, missing data, and retrospective data entry and reconciliation. A number of participants were concerned that risks and incidents may emerge in future:

*“It’s a huge burden that people have to carry because we don’t know what wasn’t done. So, we can only hope that we captured all of the patients that weren’t seen that need to be seen. We don’t…We can’t be certain. We don’t have any kind of procedures in place to be able to follow this through, which is a very unnerving place to be when you’re responsible for the health of a patient and particularly where time can be of grave importance in terms of outcomes”.* (Participant M3)

Staff wanted reassurance that the health system has learnt from this experience and will be able to better protect staff and patients from the impact of a similar event should it occur in the future. One participant working in an acute setting asked *“what has been put in place nationally to help protect us from this and protect our patients from something similar”* and further stated *“because it’s likely it’s going to come again, we need to be more prepared”.* (Participant A7).

## Discussion

While determinations on health system resilience often focus on surges in demand due to natural disasters and disease outbreaks, shocks that do not directly impact demand but compromise the ability of a national system of healthcare to deliver core services are rarely explored. The ransomware cyber-attack in May 2021 on the national ICT infrastructure of the public health system in Ireland resulted in the widespread removal of access to ICT systems across all public health services. Healthcare services are especially vulnerable to cyber-attacks due to the nature of services, where loss of access to electronic health records, radiology, and pathology results can have a devastating impact on patient safety [14, 15].

In the absence of ICT systems, staff within the Irish health service and allied service providers rapidly focused on developing and implementing manual workarounds to ensure continuity of services and maintain patient safety. Experienced staff assumed leadership roles and offered reassurance and guidance to colleagues who had no experience operating outside of a digital environment. The knowledge and experience of these staff members ensured that risks to patient safety were minimised [8]. Psychological safety is important in healthcare, particularly in challenging times. This study finds that staff felt empowered to speak-up, respond to the challenges and introduce local adaptions to ensure continuity of service. This is reflective of a service that prioritises patient safety, a supportive environment, familiarity with colleagues, and the flattened hierarchy [16]. Transparent communication and good collaboration are both key strategies to building a resilience health system [17]. Psychological safety promotes collaborative practices, which in turn supports quality and safe care [18]. Staff collaborated and recognised the importance of teamwork and effective communication needed to introduce the necessary mitigations. An independent report carried out to determine the facts surrounding the cyber-attack [6] noted the dedication and effort by individuals at all levels from across the HSE, impacted hospitals, CHOs, and third parties all going “above and beyond” in their call of duty in response to this incidence. The report noted that, in times of significant challenge or emergencies, staff in the health services are resilient, respond quickly, and have an ability to implement actions and workarounds to maintain even a basic continuity of service to their patients.

However, the effect the cyber-attack had on those working within the Irish health system cannot be underestimated. While the resilience, dedication, and innovation of staff ensured necessary services remained open, the findings further comment that this environment put additional stress on a system of healthcare professionals that were already exhausted by four waves of the COVID-19 pandemic. While the pandemic has severely exacerbated workplace stress for healthcare workers worldwide [19], participants in this study felt the stress placed on staff by the attack was more severe than the cumulative effect of the pandemic. This stress was further amplified by a lack of public awareness about the impact and duration of the cyber-attack. While there was no evidence that healthcare provision in the immediate aftermath of the attack has resulted in harm to patients, many staff carry the burden of worry over clinical decisions made and the potential loss of patient information during this period.

Healthcare quality is the key outcome for resilience in healthcare [20]. Healthcare quality includes clinical effectiveness, patient safety, timeliness, patient centeredness, care coordination, efficiency, and equity [21], and each of these dimensions was compromised during the cyber-attack. For example, equity, patient centredness, and timeliness were all compromised by the health systems inability to withstand this attack and to resume normal service speedily, further increasing already long waiting lists. Resilience must be designed within a health system. Robust, self-regulating health systems need investment in the so-called slow variables, ones that take a long time to change but are required to construct a stable platform for health care delivery [4]. National leadership, a committed workforce and sufficient infrastructure are key to building resilience [22]. Preparedness is key for resilience [17], yet there is little evidence that building resilience was a priority within the Irish health system. There was no assessment of system capacities and weaknesses, and no investment in vulnerable components of the system [6]. Resilient performance is achieved through a combination of absorption of challenges, adaptation and transformation to continue operations in the face of disruptions [23]. Those working within the Irish health system absorbed the challenge of the response to the cyber-attack and introduced adaptions to keep services functioning, at least at a basic level. However, adaptions were largely temporary, and transformation could not occur due to the system’s lack of readiness for such an event. The dedication, innovation, and commitment of health workers propped-up the health system at this time of crisis. This is resilience by happenstance rather than design [4].

### Limitations

This study is subject to certain limitations. Focus groups explored the experiences of staff working in acute, maternity, and community settings and staff from a variety of services participated, however their experiences may not reflect those of staff working within other regions of the national public health service. The experiences of health service staff working at a national level, including those working within IT who were greatly impacted by the cyber-attack, are not captured in this study. Also, while groups of four to six participants are often considered optimal in health services research [24], two of the eight groups in this study had just two participants, while a further two had three participants. Participants consisted of busy frontline staff, working in an environment that was recovering from the dual effects of the cyber-attack and covid. More participants were invited, and flexibility offered around scheduling of the focus groups but not all were available as patient care took priority. Furthermore, while focus group transcripts were made available during the publication process for this manuscript, ethical approval was sought on the basis that transcripts would be destroyed two years following the focus groups. Finally, this study was conducted in September/October 2021, shortly after the cyber-attack, and does not reflect staff experiences of operational and infrastructural IT system improvements subsequently introduced.

## Conclusion

The cyber-attack on the Irish health system in May 2021 resulted in the widespread removal of access to ICT systems. While services including radiology, diagnostics, maternity, and oncology were prioritised for reinstatement, recovery efforts continued for over four months, a timescale far greater that initially expected. The absence of the appropriate capacity within the health system infrastructure to protect patients, service users, and staff from the impact of such an attack, meant this lack of system resilience was compensated for by a resilient workforce. Within the context of the prevailing COVID-19 pandemic, this was an enormous burden on a dedicated workforce. The adverse impact of this attack may have long-term and far-reaching consequences for the wellbeing of staff. Design and investment in a resilient health system must be prioritised.

### Electronic supplementary material

Below is the link to the electronic supplementary material.

Supplementary Material 1

## Data Availability

The datasets generated and analysed during the current study are not publicly available as ethical approval was granted on the basis that the anonymised transcripts would be held by the HSE for 2 years then securely destroyed.
