# Supplementary material for: A resilient workforce: patient safety and the workforce response to a cyber-attack on the ICT systems of the national health service in Ireland
Source: BMC Health Serv Res. 2023 Oct 17;23:1112. doi: 10.1186/s12913-023-10076-8 (PMC10583305; doi:10.1186/s12913-023-10076-8)
Supplement: Supplementary file 1 — Supplementary Material 1 [file 12913_2023_10076_MOESM1_ESM.docx]

**Appendices**

**Appendix File 1:** **Study information sheet**


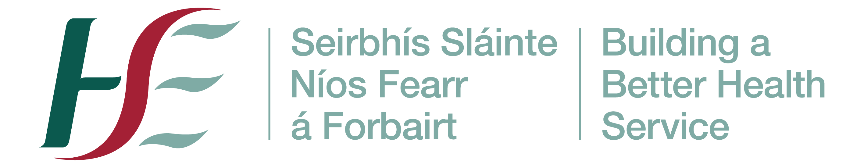


**PARTICIPANT INFORMATION SHEET**

**Study Title:** A mixed methods analysis of the effectiveness of the Patient Safety Risk Mitigation strategies following a Healthcare Information Communication Technology failure

**Principal Investigators:** Dr Orla Healy^[[1]](#footnote-1)^

**Researchers:** Dr Gemma Moore^[[2]](#footnote-2),^ Ms. Zuneera Khurshid^[[3]](#footnote-3)^

You are kindly invited to take part in this research to explore the risk mitigation measures and contingencies required to safely deliver patient services and maintain health and social care services in the event of an either partial or complete Information Communication Technology outage and to understand what worked well and where is there scope for improvement. The research is being conducted by the Quality and Patient Safety Division of the HSE. Before you make your participation decision, we would like you to understand why the research is being conducted and what it would involve for you. Please take your time to read this information. You can ask for any clarification or further information by contacting us using the details at the end of this information sheet.

**Information About This Study**

**What is this research and why is the research being done?**

In May 2021, HSE Information Communication Technology systems were the subject of a ransomware attack that resulted in widespread outages of critical Information Communication Technology systems. The objective of this study is to conduct a timely review of the mitigations and contingencies adopted in the Irish health system to minimize the impact on patient safety. In addition, the study will focus on the learning regarding what worked well to inform future planning for further Information Communication Technology outages. We are now seeking your consent to take part in a voluntary interview or focus group for the study. Team members will collectively decide whether they wish to participate in individual interviews or one focus group for the entire team. This interview will take approximately 30-40 minutes and will be conducted over the phone or as an online meeting depending on your preference. The focus group will be 1 hour long and conducted using an online meeting platform. Interviews and focus groups will be audio recorded, and pseudonyms will be used in the research reports. You have the right to withdraw from participation in the interview at any point.

**Why have I been asked to take part?**

You have been invited to take part in the research because we want to explore the experience of staff working in acute and community settings impacted by the cyber-attack.

**Do I have to take part?**

Participation in this study is entirely voluntary. It is up to you to decide whether you would like to take part or not. If you agree to take part, we will ask you to sign a consent form. You are free to refuse to take part, or to withdraw at any point, without giving a reason and without any adverse effects as a finding.

**What will happen if I agree to take part?**

If you agree to take part, the research team will ask you to sign a consent form. By opting into the study, you will be sharing your experiences of what mitigations were successful or unsuccessful from a patient safety perspective and the key learning to minimise the impact on patient safety in the case of an IT outage in the future. You are also agreeing to be contacted by the research team by email/ phone (based on your personal contact preference) about your engagement in the research.

**Will I receive any expenses or payments?**

We will not provide any payments or cover any expenses for your participation in this research.

**What are the possible risks of taking part?**

We do not envisage any harm to participants due to their participation in the research. If you decide to take part in the study, you are free to withdraw any time without question or reason. Should any information come to light during the research that would suggest malpractice or misconduct or indicate that any individual was in danger of harm; the researchers are obliged to report this to the appropriate personnel.

In the unlikely event during data collection and analysis that the researchers interpret a situation as presenting an on-going serious safety concern in respect of service provision and/or posing a clinical risk, the researcher will, where possible, bring this to the attention of the participant who highlighted the issue and ask them to submit the concern or report the incident through the appropriate channels. In addition, the researcher will include reference to the said concern in the research findings report which will be reviewed by your unit prior to circulation to the hospital management team. The report findings will be in aggregate form and all respondents and data will be anonymised.

If the nature of the concern were one that could in the view of the researcher cause serious direct and severe harm to patients, the researcher would be obliged to report this to the unit manager at the earliest opportunity. Respondents would be anonymised in the said report.

**What are the possible benefits of taking part?**

Your participation in this research will enable the Irish health system to learn from staff experiences and apply this learning to inform future healthcare policy, planning and delivery. The research will also contribute towards quality and patient safety research on issues of national priority with the potential to make a real difference to the Irish health service. It will also highlight the extraordinary work being undertaken Irish the healthcare staff to ensure service continuity during this cyber-attack.

**What will happen if I change my mind about taking part?**

If you agree to take part but later decide that you wish to withdraw, please contact a member of the research team using the contact details at the end of this leaflet. If you withdraw your consent during the study, you will not have to continue to take part in the study.

**Will my taking part in the study be kept confidential?**

Your name will not appear in reports, publications or presentations arising from the research. In accordance with HSE’s policy on data protection and storage, the paper versions of consent forms will be anonymised (your name and any identifying details will not be included) and will be kept in a locked filing cabinet in the HSE. These will only be available to members of the research team. Interviews will be transcribed and securely stored on password protected computers. Following transcription, taped recordings will be destroyed. The anonymised transcripts will be held by the HSE for 2 years then securely destroyed.

**What will happen to the findings of the study?**

The findings of this study will inform future planning to minimize the impact on patient safety of Information Communication Technology outages at local and national level. We intend to publish the findings in reports, scientific journals and to present at national and international conferences.

**Who is organising and funding the research?**

The research is being conducted by the Quality and Patient Safety Division of the HSE.

**Who has reviewed the study?**

This study has received favourable ethical opinion by Clinical Research Ethics Committee of the Cork Teaching Hospitals.

**How will I find out what happens with this project?**

If you would like to receive a summary of the findings, you can notify the researcher that you would like to be contacted for this purpose.

**What happens next?**

If you are happy to proceed, we will explain the study and answer any questions you may have.

**How to contact us?**

If you have any concerns about the study, or would like more information, please contact:

Dr. Gemma Moore PhD

Email: [gemma.moore2@hse.ie](mailto:gemma.moore2@hse.ie)

**Thank you for taking the time to read this information**

**Appendix 2: Focus group topic guide**

**Introductory questions**

1. Could I ask you **introduce** yourself and describe your **role and responsibilities** in your team?

**Impact of Cyber-attack**

1. I am interested in understanding the **impact** of the cyber-attack on how you work. What IT systems, software, and networked devices that you use were impacted by the cyber-attack?
   - How did not having access to these systems impact the services your team provides?
     - Probe further on impact on patient care, delayed procedures etc.
2. Was there effective communication to front line staff during the attack?
   - Did you feel you could effectively communicate and escalate risks and issues to clinical and executive management?
   - Were the risks you reported acted on?
     - Probe on whether they got feedback and assurance on the risks they reported

**Mitigations**

1. Could you describe the **mitigations** and workarounds that were developed to ensure continuity of services
   - How were these mitigations developed (e.g. team huddles, revision over time)
2. How did you **prioritise** the services you delivered?
   - Were there any trade-offs involved or reduction of normal practices?
3. Which of the mitigations developed worked well and which didn’t?
   - Will you retain any of mitigations in your normal routine or work practices going forward?
   - Are these mitigations being retained service wide or just within your team?
   - Who were the decision makers behind retaining the mitigations (Senior management, Local management, team?)
     - Probe on potential risks this might involve
4. Has the team and/or organisation discussed **strategies and contingency plans** for possible **future outages**?
   - Which mitigations do you plan on putting in place?

**Quality and Safety**

1. In your opinion, was there an **impact on the quality and safety of patient care** during this time?
   - Were different issues and risks more significant in certain stages of the cyber-attack? e.g. In the initial few days, while implementing workarounds or while returning to normalcy?
     - Probe on whether they are aware of any lost data during the down time
     - Probe on impact on backlogs, wait times, communications between departments, delays,
2. Are you aware of any **actual** **incidents** or errors that occurred during this time that can be attributed to the cyber-attack?
   - Were any of these serious incidents or errors that caused harm?
3. Did you think the cyber-attack influenced the levels of **stress and fatigue** among your team?

**Summary**

1. What is the current status and expectations about resumption of full services?
   - Probe on whether they are aware of any lost data during the down time
2. What is the **key learning** for the team from this experience?

**Appendix 3:** Focus group consent form


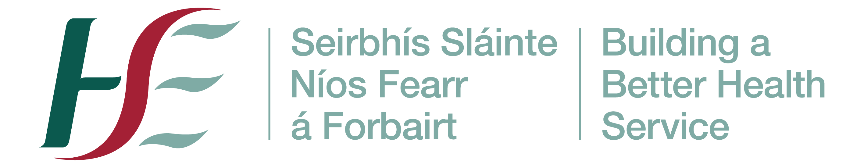


**PARTICIPANT CONSENT FORM**

**Study Title:** A mixed methods analysis of the effectiveness of the Patient Safety Risk Mitigation strategies following an Information Communication Technology failure

**Principal Investigator:** Dr Orla Healy^[[4]](#footnote-4)^

**Researchers:** Dr Gemma Moore^[[5]](#footnote-5),^ Ms. Zuneera Khurshid^[[6]](#footnote-6)^

**Participant Number:**

**Please tick each**

| 1. I have read the information sheet and understand that I will be involved in this research to explore the effectiveness of the Patient Safety Risk Mitigation strategies following a Healthcare Information Communication Technology failure |  |
| --- | --- |
| 2. I understand that my participation in this study is voluntary and that I am free to withdraw my participation at any time without giving a reason. |  |
| 3. I understand that I will be taking part in a 1 hour long focus group with a member of the research team, but that this is voluntary, and I can decline to take part if I wish. If I choose to take part, I know I can withdraw at any point up to or during the interview and can receive a copy of my transcript for my review after the interview. |  |
| 4. I understand that all data collected during the study will remain confidential, and I consent to my responses and personal information being stored in password protected and encrypted computers. |  |
| 5. I understand that if any disclosures are made that would indicate malpractice or misconduct at any point during the study or suggest that any individual was in danger of harm, this information will be disclosed to the appropriate personnel and the researcher would be obliged to report this to the unit manager at the earliest opportunity. |  |
| 6. My queries have been addressed to my satisfaction by the research team and I consent to take part in this study. |  |

Name of participant Date Signature

1. HSE National Clinical Lead for Patient Safety and Adjunct Clinical Professor in the Department of Epidemiology and Public Health in UCC [↑](#footnote-ref-1)
2. Qualitative Data Lead, Evidence for Improvement, National Quality Improvement Team, HSE [↑](#footnote-ref-2)
3. PhD Candidate, University College Dublin [↑](#footnote-ref-3)
4. HSE National Clinical Lead for Patient Safety and Adjunct Clinical Professor in the Department of Epidemiology and Public Health in UCC [↑](#footnote-ref-4)
5. Qualitative Data Lead, Evidence for Improvement, National Quality Improvement Team, HSE [↑](#footnote-ref-5)
6. PhD Candidate, University College Dublin [↑](#footnote-ref-6)
